# The Relative Association of Collective Efficacy in School and Neighborhood Contexts With Adolescent Alcohol Use

**DOI:** 10.2188/jea.JE20180125

**Published:** 2019-10-05

**Authors:** Minoru Takakura, Masaya Miyagi, Masaru Ueji, Minoru Kobayashi, Atsushi Kurihara, Akira Kyan

**Affiliations:** 1 Faculty of Medicine, University of the Ryukyus, Okinawa, Japan; 2 Faculty of Education, University of the Ryukyus, Okinawa, Japan; 3 Faculty of Education, Ibaraki University, Ibaraki, Japan; 4 Graduate School of Education, University of the Ryukyus, Okinawa, Japan; 5 Faculty of Education, Saga University, Saga, Japan; 6 Graduate School of Health Sciences, University of the Ryukyus, Okinawa, Japan

**Keywords:** social process, alcohol consumption, high school students, multilevel model, Japan

## Abstract

**Background:**

It is unclear whether either neighborhood collective efficacy or school collective efficacy is associated with adolescent alcohol use. This study aimed to examine the relative contributions of collective efficacy, both in school and in the neighborhood contexts, to alcohol use among Japanese adolescents.

**Methods:**

A cross-sectional study was conducted in public high schools across Okinawa and Ibaraki Prefectures in Japan in 2016. The study participants consisted of 3,291 students in grades 10 through 12 cross-nested in 51 schools and 107 neighborhoods. Alcohol use was measured as current alcohol drinking, which was defined as self-reported drinking on at least 1 day in the past 30 days. Collective efficacy was measured using scales of social cohesion and informal social control in school and the neighborhood. Contextual-level collective efficacy was measured using aggregated school-level and neighborhood-level individual responses, respectively. We used non-hierarchical multilevel models to fit the cross-nested data.

**Results:**

Significant variation in alcohol use was shown between schools but not between neighborhoods. After adjusting for covariates, school collective efficacy at individual- and contextual-levels was protectively associated with alcohol drinking (odds ratio [OR] for the increase of one standard deviation from the mean 0.72; 95% confidence interval [CI], 0.63–0.82 and OR 0.61; 95% CI, 0.49–0.75, respectively), whereas neighborhood collective efficacy at individual- and contextual-levels was not associated with alcohol consumption.

**Conclusion:**

The school-level associations of collective efficacy with adolescent alcohol use may have the greater impact than the neighborhood-level associations. Adolescent drinking prevention efforts should include enhancing school collective efficacy.

## INTRODUCTION

Alcohol use among young people is a significant public health problem given its adverse consequences to health and well-being in youth and later in life.^1^ Although the prevalence of adolescent alcohol use in many countries including Japan has been declining,^1^^,^^2^ it still remains as the main risk factor contributing to the global burden of disease in young people.^3^ Understanding the potential determinants of adolescent alcohol use is important for developing effective prevention strategies and interventions. According to ecological models of health behavior,^4^ adolescent alcohol use may be influenced by not only individual-level factors, but also by contextual- or collective-level factors. In the field of public health, it is widely considered that social processes in the groups, such as social capital and collective efficacy, seem to operate as a collective force that plays a significant role in health and health-related behaviors.^5^ As such, this study focused on collective efficacy as an important social process that is related to adolescent alcohol use.

Collective efficacy is a form of social capital^6^^–^^8^ and is defined as a combination of social cohesion and informal social control among neighbors. It reflects the linkage of mutual trust and the willingness of people to intervene for the common good, such as the prevention of crime and violence.^9^ Social cohesive neighborhoods are assumed to be the most fertile contexts for realization of informal social control.^9^ Adolescent behaviors may be more effectively regulated in a group where its members share mutual trust and norms about acceptable behaviors that enables them to mobilize resources that intend to control problem behaviors.^10^ Many empirical studies have shown that neighborhood collective efficacy was protectively associated with various health outcomes among adolescents.^7^^,^^11^^–^^14^ However, varied findings of studies on the association of collective efficacy with adolescent alcohol use were also reported. A systematic review of the influence of neighborhood-level social factors on alcohol use showed that collective efficacy was generally associated with lower alcohol use.^15^ On the other hand, another systematic review of multilevel evidence showed that most studies found no associations of collective efficacy with individual-level alcohol drinking.^16^ It has also been pointed out that adolescent health outcomes are differently influenced by not only the characteristics of their surrounding neighborhoods but also by other social settings, such as schools in which they spend substantial portions of their daytime hours.^17^^,^^18^ Therefore, further studies are needed to understand if and how collective efficacy at different contextual levels influence adolescent alcohol use.

School is an important context that may share influences and exert collective force on students’ daily life, health, and well-being.^8^^,^^19^ In the field of educational sciences, studies on collective efficacy in school and academic achievement have been often conducted.^20^ However, studies focusing on the association between collective efficacy in school and adolescent health-related behaviors are sparse.^10^^,^^21^ For example, a study among Greek students defined school collective efficacy as cohesion and trust among class members combined with their willingness to intervene in the case of bullying incidents. The multilevel modelling showed that individual-level bullying victimization was more frequent in school classes with lower levels of collective efficacy.^10^ A multilevel study of Swedish students, which measured collective efficacy by only two items of social cohesion and informal social control in school, showed that school-level collective efficacy might play a protective role in adolescent health-related behaviors, including alcohol use.^21^

Based on previous studies, either neighborhood collective efficacy or school collective efficacy has been separately found to be associated with adolescent health outcomes. However, in reality, adolescents are simultaneously exposed to neighborhood and school contexts. As neighborhoods and schools are crossed with each other, adolescents become nested in cross-classification of neighborhoods and schools. The separate multilevel study of the neighborhood context implies inadequate control for confounding effects of the school context, and vice versa.^22^ Ignoring the cross-classification within non-hierarchical data structure may cause misleading results due to over- or under-estimation of the parameters of interest.^23^^,^^24^ Thus, in order to examine the contextual effects of neighborhoods and schools on adolescent alcohol use simultaneously, the application of cross-classified multilevel models is needed.^25^ Although some studies have examined the simultaneous associations of neighborhood and school contexts on adolescent health outcomes,^19^^,^^22^^–^^24^^,^^26^^–^^30^ no study has examined the contextual effects of neighborhood and school collective efficacy on adolescent alcohol use. Therefore, this study investigated the relative contributions of school contexts and neighborhood contexts to alcohol use among Japanese adolescents and examined the simultaneous associations between collective efficacy in school and neighborhood and adolescent alcohol use.

## METHODS

### Study participants and procedures

A cross-sectional study was conducted in public high schools across Okinawa and Ibaraki Prefectures in Japan. After permission to conduct the study was obtained from the principals of the study schools, teachers distributed an anonymous self-administered questionnaire in classrooms from September to December 2016 using written instructions provided by researchers. After providing information about the purpose and the ethical considerations of the study, all students attending the class were requested to complete and return the questionnaire sealed in an unmarked envelope to ensure the confidentiality of the responses. Students were free to decline to participate in the study. Returning the questionnaire constituted informed consent. No follow-up was conducted on students absent from school when the survey was conducted. The study protocol was approved by the Institutional Review Board of the University of the Ryukyus (No. 343).

The study sample consisted of 5,600 students in grades 10 through 12 (aged 15–18 years) enrolled in 51 public high schools who agreed to participate in the study. The study schools were randomly chosen with a probability that was proportional to the number of schools within school types and school districts in each prefecture. Among the invited schools, 30 schools in Okinawa (the participation rate 100%) and 21 schools in Ibaraki (the participation rate 66%) agreed to take part in the survey. There were no differences in school characteristics (school type, area, and school size) between participating schools and non-participating schools in the survey of Ibaraki. In each school, one class was chosen from each grade, except for six schools in Ibaraki that did not include grade 12 due to the schools’ schedule. Among the study sample, a total of 5,110 students participated in this study (224 declined to participate and 266 were absent form school). The researchers excluded 18 students who did not provide information about sex, leaving 5,092 students.

The study participants were requested to provide a postal code for their place of residence. In this study, neighborhoods were defined by postal code areas reflecting the municipal level. The postal code in Japan consists of seven-digit numbers, which represent small blocks of town areas. When seven-digit postal codes were adopted by the researchers, 83% of the neighborhoods had less than five participants. Thus, we used the first five-digit postal codes, which represent large blocks of town areas, to obtain sufficient samples. We excluded those who had missing or invalid information on postal codes (*n* = 1,349 and *n* = 14, respectively). To ensure valid and reliable estimates of the neighborhood-level parameters,^30^^,^^31^ those who lived in the five-digit postal code areas that contained less than five participants were also excluded (*n* = 138 in 69 neighborhoods, which was 39% of the five-digit postal code areas). Then, 3,591 students cross-nested in 51 schools and 107 neighborhoods were retained. Finally, complete data on all variables of interest were available for 3,291 students and these data were used for analysis (64% of the original participants). There was an average of 10.5 neighborhoods per school (range, 2–21) and an average of 5.0 schools per neighborhood (range, 1–14), resulting in 538 different combinations of schools and neighborhoods. Students in the same school lived in different neighborhoods, and students form the same neighborhood attended different schools. Thus, there was non-hierarchical data structure of schools and neighborhoods. The average numbers of students per school and neighborhood were 65 (range, 17–100) and 31 (range, 5–218), respectively. A detailed flow diagram of the study participants is available in eFigure 1.

### Measures

#### Alcohol use

Alcohol use was assessed using a question adapted from the Youth Risk Behavior Surveillance conducted by the United States Centers for Disease Control and Prevention.^32^ We measured alcohol use from student’s response to the following question: “During the past 30 days, on how many days did you have at least one drink of alcohol?” A current drinker was defined as one who consumed alcohol on at least 1 day in the past month.^32^ Test-retest reliability of this question demonstrated adequate stability for Japanese adolescents, with kappa statistics of 0.51.^33^

#### Collective efficacy

Collective efficacy was measured using school and neighborhood collective efficacy scales.^8^ Each collective efficacy scale was conceptualized as a combination of social cohesion and informal social control, based on Sampson’s scale.^9^ The scales of social cohesion comprised seven items on school and five items on the neighborhood, indicating trust and reciprocity among students and teachers at school as well as with their neighbors in their neighborhoods. The scales of informal social control were composed of seven items on school and six items on the neighborhood which represent the willingness of students or neighbors to intervene in cases of trouble in school or in the neighborhood. All collective efficacy items shown in eMaterial 1 were rated using a five-point scale ranging from “strongly disagree” to “strongly agree”. The sum of the scores ranged from 14 to 70 for school collective efficacy and from 11 to 55 for neighborhood collective efficacy. The scales have been shown to have adequate reliability and validity among Japanese adolescents.^8^ In the current sample, Cronbach’s alphas of the scales ranged from 0.92 to 0.96. Contextual-level collective efficacy was measured using aggregated school-level and neighborhood-level individual responses, respectively (ie, school- and neighborhood-level mean scale scores). Preliminarily, we examined the social cohesion and informal social control separately. However, there were strong correlations between contextual-level school social cohesion and school informal social control (*r* = 0.919) as well as between contextual-level neighborhood social cohesion and neighborhood informal social control (*r* = 0.573). This leads to a strong multicollinearity in the model. In fact, the subsequent multilevel models showed distorted findings, such as a sign-reversal and small odds ratios (ORs). Therefore, we did not analyze the social cohesion and informal social control separately.

#### Covariates

Several demographic factors that are considered to be potential confounders were included as covariates. These variables were prefecture, sex, grade, school type (general or vocational), family structure, and parental education level. Family structure was based on the person with whom the student was living. The response was dichotomized as “living with both parents” or “other.” Parental education level was based on information gathered from students about their mother’s or father’s educational attainment. The higher level of education attained by either parent was included in the analysis. The categories used for analysis were “high school or less”, “specialized training college or junior college”, and “university or more”. These categories are consistent with the International Standard Classification of Education levels, 1/2/3, 5B, and 5A/6, respectively.^34^ School-level socioeconomic status was also considered as a control variable. The unemployment rates of the municipalities in which the study schools were located were included as a measure of contextual-level socioeconomic status. Data on the unemployment rates were obtained from the results of the 2015 population census.^35^

### Data analyses

Initially, descriptive statistics for the participants were shown. Bivariate statistics between alcohol use and explanatory variables were presented as crude ORs. In the remaining analyses, we conducted the analyses for the pooled sample because there was no significant interaction between sex and collective efficacy variables or between prefecture and collective efficacy variables on alcohol use. First, we estimated variations between schools or neighborhoods in alcohol use separately using standard two-level random intercept models with no explanatory variables and either schools or neighborhoods as random effects (ie, a school only multilevel model or a neighborhood only multilevel model). Next, we conducted a cross-classified multilevel model with no explanatory variables considering school and neighborhood variations simultaneously.^25^ Then, the multilevel model was extended to include covariates (model 1). Finally, we included collective efficacy variables and covariates (model 2). In order to obtain stable solutions in the models, individual-level collective efficacy variables were group-centered on their school- or neighborhood-level means.^25^ Fixed-effect estimates were converted into ORs with 95% confidence intervals (CIs). For the collective efficacy variables treated as continuous variables, ORs were computed for the increase of one standard deviation in the corresponding collective efficacy characteristics. As measures of variability (random effects), the proportion of the total variance in alcohol use attributable to schools or to the neighborhoods was estimated using the intraclass correlation coefficient (ICC), which was calculated using the latent variable method and approximating the individual variance by π^2^/3.^36^ We also calculated the median odds ratio (MOR), which indicates the median value of the OR for all possible comparisons of individuals from lower to higher drinking prevalence groups (MOR = exp[0.95 × sqrt(school- or neighborhood-level variance)]).^36^ All models were performed based on a logit-link function using the GLIMMIX procedure in SAS version 9.4 (SAS Institute, Cary, NC, USA). The estimation procedure used in this study was restricted pseudo-likelihood estimation with an expansion around the current estimate of the best linear unbiased predictors of the random effects.^37^

## RESULTS

The descriptive characteristics of the participants and prevalence of current alcohol use by demographic factors are shown in Table 1. Overall, current drinking prevalence was 9.6%. The mean prevalence of current alcohol use across schools was 10.1% (range, 0–38%), and 9.8% across the neighborhoods (range, 0–42%). There were no differences in current drinking prevalence by prefecture and family structure, but lower graders, girls, students at general high schools, and students with higher parental education had lower current drinking prevalence than other students. Collective efficacy in school and neighborhood at the individual level as well as at the contextual level were negatively associated with current alcohol use. Contextual-level unemployment was not associated with alcohol use.

**Table 1.** Characteristics of demographic factors and collective efficacy variables and prevalence of current alcohol use

		*n*	%	Current alcohol use		

				%	OR	95% CI
Total		3,291	100	9.6		
Prefecture	Okinawa	2,071	62.9	9.6	1.00	
	Ibaraki	1,220	37.1	9.6	1.00	0.79–1.28
Grade	10	1,118	34.0	6.2	1.00	
	11	1,127	34.2	8.6	**1.43**	**1.04–1.97**
	12	1,046	31.8	14.2	**2.53**	**1.87–3.41**
Sex	Boy	1,490	45.3	10.7	1.00	
	Girl	1,801	54.7	8.6	**0.78**	**0.62–0.99**
School type	General HS	2,362	71.8	7.0	1.00	
	Vocational HS	929	28.2	16.1	**2.56**	**2.03–3.25**
Family structure	Both parents	2,475	75.2	9.1	1.00	
	Others	816	24.8	10.9	1.22	0.94–1.58
Parental education	JHS/HS	1,385	42.1	12.8	1.00	
	Spec/college	856	26.0	8.9	**0.66**	**0.50–0.88**
	University or more	1,050	31.9	5.9	**0.43**	**0.32–0.58**

		Mean	SD		OR^a^	95% CI
Contextual-level unemployment (%) (*n* = 51)^b^		5.39	1.20		0.98	0.87–1.10
Individual-level	School collective efficacy	48.3	10.9		**0.58**	**0.51–0.65**
	Neighborhood collective efficacy	38.4	8.9		**0.72**	**0.65–0.81**
Contextual-level	School collective efficacy (*n* = 51)	47.8	4.7		**0.84**	**0.79–0.90**
	Neighborhood collective efficacy (*n* = 107)	38.5	2.7		**0.91**	**0.88–0.94**

CI, confidence interval; HS, high school; JHS, junior high school; OR, odds ratio.

Bold ORs are statistically significant (*P* < 0.05).

^a^ORs are computed for the increase of 1 standard deviation.

^b^The unemployment rate of the municipality in which the school is located.

Table 2 shows random-effects estimates for the school-only, neighborhood-only, and cross-classified multilevel models without any explanatory variables. Variation in current alcohol use between schools in the school-only model was 0.586 (standard error [SE], 0.169; *P* = 0.001) and between-neighborhood variance in the neighborhood-only model was 0.100 (SE, 0.078; *P* = 0.204). The ICCs obtained for the school-only and neighborhood-only model were 15% and 3%, respectively. In other words, not considering other contexts, about 15% of the variation in alcohol use was attributable to differences between schools and 3% was due to differences between neighborhoods. The MORs were 2.1 at the school level and 1.3 at the neighborhood level. In the null cross-classified model considering school- and neighborhood-level random effects simultaneously, between-school variance had a similar value with the school-only model (σ^2^ = 0.586; SE, 0.169; *P* = 0.001), but the random effect at the neighborhood level was not definitively positive, which indicated that between-neighborhood variance was estimated to be zero. Therefore, we removed the between-neighborhood variance from random parameter estimations and used standard two-level multilevel models with schools as random effects in the subsequent models.

**Table 2.** Measures of variations in current alcohol use base on multilevel models without any explanatory variables (*n* = 3,291)

Random effect parameters	School-only MM					Neighborhood-only MM					CCMM				
		
	σ^2^	(SE)	*P*	ICC	MOR	σ^2^	(SE)	*P*	ICC	MOR	σ^2^	(SE)	*P*	ICC	MOR
School-level variance	0.586	(0.169)	0.001	15.1	2.1	—					0.586	(0.169)	0.001	15.1	2.1
Neighborhood-level variance	—					0.100	(0.078)	0.204	2.9	1.3	0.000				

CCMM, cross-classified multilevel model; ICC, intraclass correlation coefficient; MM, 2-level multilevel model; MOR, median odds ratio; SE, standard error.

Table 3 shows results from the multilevel model that examined the associations of individual-level and contextual-level collective efficacy and covariates with adolescent alcohol use. Findings of model 1, which included covariates, were almost in the same direction as those of bivariate analysis. The school-level variance was reduced by 49% after including covariates (σ^2^ = 0.297; SE, 0.110; *P* = 0.007). In model 2, adding collective efficacy variables, individual-level school collective efficacy was associated with adolescent drinking (OR 0.72; 95% CI, 0.63–0.82). Similarly, contextual-level school collective efficacy was also associated with adolescent drinking (OR 0.61; 95% CI, 0.49–0.75). Meanwhile, neighborhood collective efficacy variables both in individual- and contextual-levels were not associated with drinking. After including all explanatory variables, the school-level variance in drinking was attenuated (σ^2^ = 0.164; SE, 0.075; *P* = 0.029), which means that collective efficacy variables and covariates accounted for 72% of the variability between schools.

**Table 3.** Associations of collective efficacy variables and covariates with current alcohol use by the multilevel model (*n* = 3,291)

**Fixed effect parameters**	Model 1				Model 2			
	
	OR	95% CI			OR	95% CI		
Prefecture								
Okinawa	1.00				1.00			
Ibaraki	1.04	0.60–1.80			0.89	0.55–1.45		
Grade								
10	1.00				1.00			
11	1.38	0.99–1.92			1.28	0.92–1.79		
12	**2.36**	**1.73–3.21**			**2.40**	**1.75–3.28**		
Sex								
Boy	1.00				1.00			
Girl	0.81	0.63–1.05			**0.76**	**0.59–0.98**		
School type								
General HS	1.00				1.00			
Vocational HS	**2.44**	**1.61–3.71**			**1.52**	**1.01–2.27**		
Family structure								
Both parents	1.00				1.00			
Others	1.00	0.76–1.32			0.95	0.72–1.25		
Parental education								
JHS/HS	1.00				1.00			
Spec/college	0.79	0.59–1.07			0.84	0.62–1.13		
University or more	**0.58**	**0.42–0.80**			**0.62**	**0.45–0.86**		

Contextual-level unemployment^a,b^	0.93	0.70–1.24			1.04	0.81–1.34		

**Individual-level**								
School collective efficacy^a^					**0.72**	**0.63–0.82**		
Neighborhood collective efficacy^a^					0.88	0.77–1.01		
**Contextual-level**								
School collective efficacy^a^					**0.61**	**0.49–0.75**		
Neighborhood collective efficacy^a^					1.01	0.86–1.19		

**Random effect parameters**	σ^2^	(SE)	ICC	MOR	σ^2^	(SE)	ICC	MOR
School-level variance	**0.297**	**0.110**	**8.3**	**1.7**	**0.164**	**0.075**	**4.7**	**1.5**

CI, confidence interval; HS, high school; ICC, intraclass correlation coefficient; JHS, junior high school; MOR, median odds ratio; OR, adjusted odds ratio; SE, standard error.

Bold ORs are statistically significant (*P* < 0.05).

^a^ORs are computed for the increase of 1 standard deviation.

^b^The unemployment rate of the municipality in which the school is located.

## DISCUSSION

This study examined the contextual effects of school and neighborhood on Japanese adolescent alcohol use and whether collective efficacy in school and neighborhood were simultaneously associated with adolescent drinking.

The result of the cross-classified multilevel model with no explanatory variables suggested that the school context explained more variation in Japanese adolescent alcohol use than the neighborhood context. This finding is consistent with previous studies using the null cross-classified model, which showed that the relative contributions of schools were greater than the neighborhoods to the variances in various health outcomes among adolescents, such as smoking,^23^^,^^24^ marijuana use,^27^ physical activity,^30^ body mass index (BMI),^29^ and depressive symptoms.^26^ For adolescent alcohol use, although there are no studies using the cross-classified model, a three-level multilevel study of Swedish students nested within schools and schools nested within city districts also showed significant variations in adolescent drinking between schools but not between city districts.^38^ Given the findings from our study and previous relevant studies, the school may be more important than the neighborhoods to explain contextual variations in adolescent alcohol use. As some researchers have pointed out, it is plausible that neighborhood-level effects are a consequence of unmeasured confounding.^24^

We also found that, in the multilevel model that included explanatory variables, school collective efficacy in individual- and school-levels were significantly and protectively associated with adolescent alcohol use. This suggests that not only students who feel social cohesion and informal social control in schools but also students in schools characterized by high aggregate levels of school collective efficacy were less likely to engage in alcohol consumption. On the other hand, neighborhood collective efficacy variables in individual- and neighborhood-levels were not associated with adolescent drinking. For school collective efficacy, our finding is in line with a two-level multilevel study that has shown that high school-level collective efficacy was associated with a lower risk of adolescent alcohol use.^21^ Meanwhile, a systematic review of multilevel evidence of neighborhood effects on adolescent alcohol use found no associations of neighborhood-level collective efficacy with adolescent alcohol use.^16^ These findings are expected because there are significant variations in adolescent drinking between schools but not between neighborhoods. In addition, since students spend most of their daytime hours in schools, they can more frequently interact with peers and teachers, experience mutual trust and shared expectations, and also learn health promoting skills in schools.^39^ Schools also have opportunities to monitor student behaviors and intervene with those at risk.^26^ Therefore, schools may exert more collective force to prevent adolescent drinking when compared to neighborhoods. Furthermore, a previous study using a cross-classified model to examine associations between school- and neighborhood-level social environments and student BMI showed that school-level connectedness was associated with lower BMI, but neighborhood-level social ties had no association with BMI.^40^ Thus, while the ORs associated with school and neighborhood collective efficacy are not directly comparable in this study due to their different scales, these findings support that school-level associations may have greater impact than neighborhood-level associations with adolescent health outcomes, including alcohol use.

The present study is the first cross-classified multilevel study examining the role of collective efficacy in school and neighborhood contexts in Japanese adolescent alcohol use. Nonetheless, several limitations should be noted. First, similar to previous cross-classified multilevel studies based on school-based surveys,^23^^,^^24^^,^^26^^,^^27^ the number of students per neighborhood was smaller than that per school. Even though we excluded neighborhoods with less than five participating students from the analysis, this may lead to a lack of statistical power in neighborhood-level effects. Second, we defined the neighborhoods as relatively large residential areas using postal codes. As smaller spatial units are more appropriate to capture associations between contextual factors and health,^41^ our neighborhood-level effects may be measured at a unit that is too large to find meaningful effects. Furthermore, our definition of neighborhoods may not coincide with how adolescents perceived their neighborhoods. Third, the study participants were exclusively from public high schools in two prefectures. According to Japanese and prefectural governments, the percentages of students attending public schools throughout Japan was 69% in 2016, while those of Okinawa and Ibaraki were 86% and 73%, respectively. This means that our study areas are not representative of Japan. Although the respondents represented the target population well in terms of gender, grade, and school type, the generalizability of the present findings to adolescents in Japan as a whole may be limited. Moreover, our data had a large amount of missing data on postal codes. Although current drinking prevalence of the participants did not differ from that of those who were excluded due to missing postal codes, collective efficacy variables’ scores in the participants were higher than those in excluded participants (data not shown). Thus, it may be that data are not missing completely at random and can potentially bias the results. Fourth, current alcohol use was assessed using a self-reported single question. Although the question’s reliability has been confirmed, it is unclear whether the question most successfully measured current alcohol use. This issue might induce misclassification or underestimation of adolescent drinking behavior in this study. Fifth, potential confounding not considered in this study may be a limitation. For example, adolescent social networks could relate to social tie connections, which influence adolescent alcohol use.^42^ Lastly, the present data were obtained cross-sectionally. Clearly, this study cannot provide any information on causal relationships. As contextual effects may have delayed long-term effects,^22^ further studies are needed to examine the longitudinal effect of contextual-level collective efficacy in school and neighborhood on adolescent alcohol use.

In conclusion, the school context explained more variation in adolescent alcohol use than the neighborhood context. School collective efficacy at the individual and school levels was protectively associated with drinking, whereas neighborhood collective efficacy at the individual and neighborhood levels was not associated with drinking. This study suggests that the school-level associations with adolescent drinking may have greater impact than the neighborhood-level associations, and that school collective efficacy is important to prevent adolescent drinking. Therefore, the school may be a promising context to more effectively address adolescent drinking prevention interventions and policies. Specifically, adolescent drinking prevention efforts should include enhancing school collective efficacy.

## APPENDIX A. SUPPLEMENTARY DATA

Appendix. School and neighborhood collective efficacy scale items

Figure 1. Flow diagram of the study participants
