# Supplementary material for: The Relative Association of Collective Efficacy in School and Neighborhood Contexts With Adolescent Alcohol Use
Source: J Epidemiol. 2019 Oct 5;29(10):384–90. doi: 10.2188/jea.JE20180125 (PMC6737185; doi:10.2188/jea.JE20180125)
Supplement: Supplementary file 1 [file je-29-384-s001.pdf]

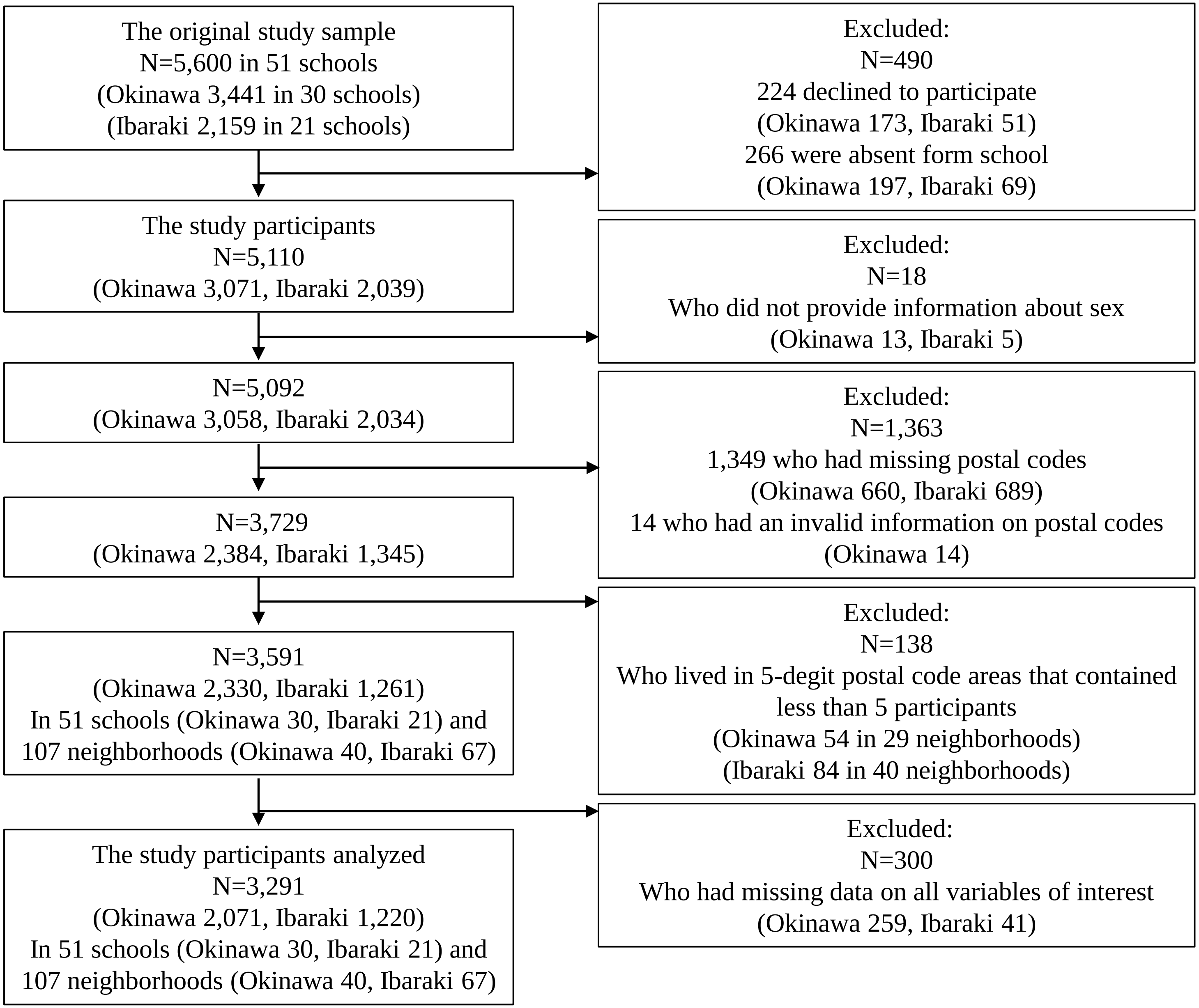

eFigure 1. Flow diagram of the study participants

eMaterial 1. School and neighborhood collective efficacy scale items

School collective efficacy

Social cohesion in school

- Students in my school are kind and dependable
- Students in my school can be trusted
- Students in my school help each other
- Students in my school usually try to be helpful
- Students in my school understand each other
- Teachers in my school are kind and dependable
- Teachers in my school can be trusted

Informal social control in school

- If classmates were drinking alcohol beverages, students in my school would do something about it
- If classmates were smoking cigarettes, students in my school would do something about it
- If classmates were skipping school and hanging out on a street corner, students in my school would do something about it
- If classmates were showing disrespect to teachers, students in my school would scold the classmates
- If classmates were making some noise during class and disturbing class, students in my school would do something about it
- If classmates were bullying someone, students in my school would do something about it
- Students in my school would work together to solve our problems

Neighborhood collective efficacy

Social cohesion in the neighborhood

- The neighbors are kind and dependable
- The neighbors help each other
- The neighbors can be trusted
- The neighbors usually try to be helpful
- The neighbors get along with each other

Informal social control in the neighborhood

- If students were smoking cigarettes, people in my neighborhood would do something about it
- If students were drinking alcohol beverages, people in my neighborhood would do something about it
- If students were showing disrespect to adults, people in my neighborhood would scold the classmates
- If students were skipping school and hanging out on a street corner, people in my neighborhood would do something about it
- People in my neighborhood would get together to improve security in the area
- People in my neighborhood would get together to improve the image of the neighborhood
